# Gender Differences in Fat-Rich Meat Choice: Influence of Personality and Attitudes

**DOI:** 10.3390/nu12051374

**Published:** 2020-05-11

**Authors:** Sara Spinelli, Caterina Dinnella, Federica Tesini, Alessandra Bendini, Ada Braghieri, Cristina Proserpio, Luisa Torri, Nicoletta A. Miele, Eugenio Aprea, Agata Mazzaglia, Tullia Gallina Toschi, Erminio Monteleone

**Affiliations:** 1 Department of Agricultural, Food, Environment and Forestry (DAGRI), University of Florence, 50144 Florence, Italy; caterina.dinnella@unifi.it (C.D.); erminio.monteleone@unifi.it (E.M.); 2 Alma Mater Studiorum, University of Bologna, 40126 Bologna, Italy; federica.tesini@unibo.it (F.T.); alessandra.bendini@unibo.it (A.B.); 3 School of Agricultural, Forest, Food, and Environmental Sciences, University of Basilicata, Via dell’Ateneo Lucano 10, 85100 Potenza, Itay; ada.braghieri@unibas.it; 4 Department of Food, Environmental and Nutritional Sciences (DeFENS), University of Milan, 20133 Milan, Italy; cristina.proserpio@unimi.it; 5 Sensory and Consumer Science, University of Gastronomic Sciences, Piazza Vittorio Emanuele II, 9, 12042 Pollenzo, Italy; l.torri@unisg.it; 6 Department of Agricultural Sciences, University of Naples Federico II, 80055, Portici, Italy; nicolettaantonella.miele@unina.it; 7 Center Agriculture Food Environment, University of Trento/Fondazione Edmund Mach, Via E. Mach 1, 38010 San Michele All’adige, Italy; eugenio.aprea@unitn.it; 8 Dipartimento di Agricoltura, Alimentazione e Ambiente (Di3A), University of Catania, Via Santa Sofia 100, 95123 Catania, Italy; agata.mazzaglia@unict.it

**Keywords:** fat preference, gender, meat, personality traits, PROP

## Abstract

The innate liking of fats may be due to one or more orosensory, post-ingestive, and metabolic signals; however, individuals differ in their preference for fat in meat. One of the variables that mainly impacts eating behaviors and thus should be carefully analyzed is sex/gender, and while sex (female/male, in a binary approximation) refers only to biological characteristics, gender (woman/man, in a binary approximation) refers to cultural attitudes and behavior. This study aimed at exploring the role of gender, age, taste responsiveness (measured as sensitivity to the bitterness of 6-n-propylthiouracil (PROP)), personality traits, attitudes, and liking of and familiarity with meat on the choice of fat-rich meat products in 1208 women and men aged 18–66. Both a between- and a within-gender approach were adopted. Results showed that gender had a major impact on liking of and familiarity with meat and choice for fat-rich meat compared to age. A lower liking meat in general was found in women, independently of fat content. Women also reported a lower familiarity than men with fatty meat and cold meat and a lower choice of fat-rich meat. Genders differed in the influence of personality and attitudes about fat-rich meat choice. In both genders, the choice of meat higher in fat was associated with liking cold and fatty meat and with age and negatively with liking low-fat meat. Women were in general more interested in health than men, and this may explain the main difference in the choice of fat-rich meat between genders. However, when we look at each gender separately, general health interest was significantly correlated with a lower choice of fat-rich meat only in men. In addition, in men food neophobia was negatively correlated with choice of fat-rich meat. In women, the emotional dimension was found to play an important role, with sensitivity to disgust that was negatively associated with fat-rich meat choice and emotional eating that was positively associated with it. Thanks to the large sample and the gender-sensitive approach adopted, this study showed that different factors affect choice of fat-rich meat by gender, in addition to liking of and familiarity with fat-rich and cold meat and age. This suggests that strategies personalized by gender to reinforce or activate barriers to this type of consumption may be more effective at reducing fat intake, promoting the consumption of meat lower in fat.

## 1. Introduction

It is well known that overconsumption of fat has negative health impacts [1,2] and increases the risk of incidence of diseases, such as obesity [3], diabetes [4] coronary heart disease [5], and cancer [6,7,8]. The innate liking of fats may be due to one or more of the orosensory, post-ingestive, and metabolic signals; however, it is evidenced that individuals differ in their preference for fat in meat [9,10]. The fat content of meat from most species has been mainly associated with texture, comprising tenderness and juiciness, and flavor [11].

Sex is a biological quality or classification of sexually-reproducing organisms, generally female, male, and/or intersex, according to functions that derive from the chromosomal complement, reproductive organs, or specific hormones or environmental factors that affect the expression of phenotypic traits that are strongly associated with females or males within a given species, while gender is a socio-cultural process that refers to cultural and social attitudes that together shape and sanction “feminine” and “masculine” behaviors, products, technologies, environments, and knowledges (http://genderedinnovations.stanford.edu/terms/distinct.html). Gender has been seen as a proxy for other measures, as it often “defines differences in perceived expectancies, environments, opportunities, income level, interaction with children, and experience in food selection and preparation, and many other variables in addition to genetic, hormonal and anatomical difference” [12]. On this premise, in the present study, which takes into account factors affecting liking of, familiarity with and choice of fat-rich meat, gender was considered instead of sex. Furthermore, a gender-sensitive approach was adopted with the aim of reporting similarities and difference between the genders, but also within each gender (namely, between women and men, respectively).

Gender has the largest impact on sensory response and food preferences [13] and was found to affect liking and consumption of meat and fat products, with women liking meat [14,15,16] and high-fat products [17] less than men. Furthermore, women reported more avoidance of fats from meat than men did [10,18,19], and a study across 22 countries showed that gender had the most consistent influence on fat preference in pork meat among other factors, with a greater proportion of women than men preferring pork with less fat cover [20]. Women tend to be more concerned about animal welfare compared to men [21,22,23], and this might explain gender differences in liking of meat [24]. Other factors play a role in preference for and consumption of fatty foods, ranging from sensory sensitivity to psychological traits and motives. Texture, odor, and flavor contribute to the liking of fatty foods [25]. Fatty acid taste sensitivity, defined as hypersensitivity to the taste of oleic acid, was negatively associated with greater consumption of fatty foods, specifically butter, meat, dairy, and with increasing BMI [26]. The positive relationship between 6-n-propyl-2-thiouracile (PROP) responsiveness and perception of fat was also suggested, but the results are controversial, as discussed by Tepper et al. [27]. Most studies [28,29,30,31,32,33] reported that PROP non-tasters had a lower ability to distinguish fat content and creaminess in certain fatty food, compared to those who taste PROP as more bitter. On the other hand, other studies reported that PROP responsiveness and both sensory response and preference for fat were unrelated [34,35] Furthermore, sensitivity to the taste of PROP and fatty acid taste sensitivity were found to be unrelated [26].

Greater fat preference was found to be inversely related to restrained eating and thus more an aspect of eating behavior than of personality [36]. Other studies with female students [37] and patients with diagnosed type 2 diabetes [38] confirmed that fat intake was negatively associated with restrained eating, while external eating was positively associated, and emotional eating was not significantly associated with it. On the other hand, reward sensitivity was found to be associated with fat and sweet food intake and alcohol consumption [39] and with fat and sweet food liking [40], suggesting that reward sensitivity may lead to preferential intake of foods rich in calories.

The literature on the relationship between personality and preference and choice of meat is quite limited: high levels of openness to experience (which characterizes curious, imaginative and openminded people who like new ideas) were associated with lower meat consumption in a large study in Germany [41]. In addition, studies supported that this relationship depends on meat types: openness was negatively associated with red meat consumption, whereas it was unrelated to poultry consumption and overall meat consumption; by contrast, extraversion was associated with higher consumption of each individual type of meat and more overall meat consumption [42].

These findings warrant further investigations to overcome some limitations of the previous studies, such as the small sample size and the focus on overweight and obese individuals. Furthermore, to the best of our knowledge, the role of gender has been under-investigated. It is unclear how different factors such as personality traits, attitudes, and taste responsiveness affect liking and choice of meat rich in fat in men and women. We might in fact hypothesize that a variable is very significant for a gender, but not for the other one, while some variables are relevant for both genders. This may be due to the fact that genders differ in personality traits and taste responsiveness. Analyses of similarities and differences both between and across genders have been encouraged [43]. Within-gender approaches, namely approaches in which each gender is investigated separately, have been proven to be very effective [44,45], in addition to between-gender studies.

The aim of this study was, therefore, to investigate, in a large sample, the factors affecting choice for fat-rich meats, including socio-demographics, taste responsiveness, personality traits, attitudes, liking of and familiarity with fat-rich meats, adopting a gender-sensitive approach. In addition, the study aimed at testing if PROP responsiveness significantly affected liking of and familiarity with fat-rich meats and if gender moderated this relationship.

Differences and similarities between genders were investigated through both a between-gender and a within-gender analysis. The former was used to study the effect of gender, while the latter was used to investigate each gender separately in order to highlight the importance that each variable had for each gender, pointing out the differences within women and men, respectively.

## 2. Materials and Methods

### 2.1. Participants

The subjects (*n* = 1208) who participated in the study were 58.36% women (*n* = 705), with a mean age of 35.5 (SD 12.9). The characteristics of the participants are reported in Table 1. Data were collected in 8 cities in different geographical areas of Italy (north: Trento, Milan, Pollenzo (Cuneo); central: Bologna and Florence; south: Napoli, Potenza, and Catania). Exclusion criteria were pregnancy, breastfeeding, not being born in Italy or having lived less than 20 years in Italy, and age not included in the range 18–66 years old.

Participants were recruited by means of announcements, social networks, participant universities and research centers’ websites, and national newspapers and magazines.

All testing involving human subjects was in compliance with the principles laid down in the Declaration of Helsinki, and all subjects provided informed, written consent prior to participation in agreement with the Italian ethical requirements on research activities and personal data protection (Law Decree 30.6.03 n. 196). The protocol of the study was approved by the Ethics Committee of Florence and Trieste University.

### 2.2. Overview of Data Collection

**Figure 1:**
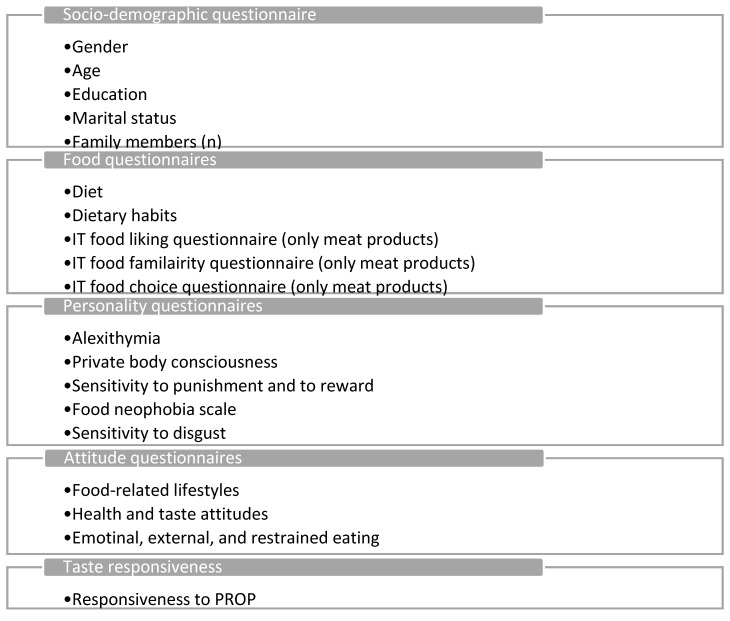
Overview of the study.

Participants were asked to fill in an online questionnaire before attending two sessions at the laboratory. Socio-demographic information (self-reported gender, age, education) and familiarity with foods were collected before the test sessions. In the lab sessions, participants were asked to fill in a set of questionnaires to measure personality and psychological traits and attitudes towards food, liking, and choice. The study included sensory tests, questionnaires, and the collection of other data (see Monteleone et al. [46] for a complete overview of data collection), but only a selection of variables will be presented here (Figure 1).

### 2.3. PROP Responsiveness

A 3.2 mM solution of 6-n-propyl-2-thiouracile (European Pharmacopoeia Reference Standard, Sigma Aldrich, Milano, IT) was prepared by dissolving 0.5447 g/L in deionized water [47]. To ensure consistency, respondents evaluated the bitter intensity of two identical samples (10 mL), presented monadically in white plastic cups and coded with two different three-digit codes [48]. Subjects were instructed to hold each sample into their mouth for 10 s, then, after expectorating, wait 20 more seconds, and subsequently, evaluate the bitterness using the Generalized Labeled Magnitude Scale (gLMS scale [49] ). In order to control the carry-over effect after the first evaluation, a break of 90 s was established between the two evaluations; during the break, respondents rinsed their mouth with water (30 s), had a cracker (30 s), then rinsed their mouth again. Mean scores between the two replicates were calculated, and participants were then grouped according to their PROP status on the basis of cut-offs reported in previous studies [46,50,51]. Respondents were classified as non-taster (NT; gLMS ≤ moderate, 17), supertaster (ST; gLMS ≥ very strong, 53), and medium taster (MT; gLMS >17 and <53).

Before the evaluation, participants were instructed on the use of the gLMS scale. Instructions were given that the top of the scale represented the most intense sensation that subjects could ever imagine experiencing. A variety of sensations from different modalities, including loudness and oral pain/irritation, were recalled to provide examples [52]. To practice the use of the scale, subjects rated intensities of the brightest light they had ever seen following the procedure described in Dinnella et al. [53]. The task was performed individually, and the criterion to conclude that the subjects correctly used the scale was that ratings must have been higher than “very strong”, but lower than “the strongest imaginable”. In the case of ratings out of this range, a short individual interview was carried out to understand the reason for the ratings, and the use of the scale was explained again. In a limited number of cases when subjects were unable to use the scale properly even after the second explanation, their evaluations were discarded from the analysis.

### 2.4. Psychological and Personality Traits

#### 2.4.1. Toronto Alexithymia Scale

Alexithymia is a construct characterized by the difficulty identifying subjective emotional feelings and distinguishing between feelings and the bodily sensations of emotional arousal, difficulty describing feelings to other people, an impoverished fantasy life, and a stimulus-bound, externally oriented cognitive style. The trait was measured using the Toronto Alexithymia Scale (TAS-20) questionnaire, structured into 3 domains: DIF, difficulty identifying feeling; DDF, difficulty describing feeling; EOT, externally oriented thinking. The questionnaire included a total of 20 items evaluated on a 5-point Likert scale (1 = disagree strongly, 7 = agree strongly) [54], validated in Italian by Bressi et al. [55]. The individual score for each domain corresponded to the sum of ratings (ranging from 20 to 100), with higher scores indicating a greater level of alexithymia reflected in the lower capabilities of identifying feelings (DIF, range 7–35), describing feelings (DDF, range 5–25), and externally-oriented thinking (EOT, range 8–40).

#### 2.4.2. Private Body Consciousness

The disposition to focus on internal bodily sensations (awareness of internal sensations) was quantified using the 5 item Private Body Consciousness (PBC) questionnaire [56], validated in Italian by Spinelli et al. [45] on a 5-point scale ranging from 1 = extremely uncharacteristic to 5 = extremely characteristic. The final individual score for PBC was the sum of the scores (ranging from 5 to 25).

#### 2.4.3. Sensitivity to Punishment and Sensitivity to Reward

This Sensitivity to Punishment and Sensitivity to Reward Questionnaire (SPSRQ) was used for the responsiveness of the two brain systems that control the behavioral activation system (BAS) and the behavioral inhibition system (BIS) [57], validated in Italian by Spinelli et al. [45]. The questionnaire included two scales: the sensitivity to punishment scale (SP) was built with items that reflected situations related to individual differences in reaction and responsiveness to BIS, while the sensitivity to reward scale (SR) was represented by items that measured the BAS functionality dealing with specific rewards (i.e., money, praising, social power). Each scale was rated with a yes/no format, and the total score for each subject was represented by the sum of “yes” answers. Based on Spinelli et al. [45], we removed 7 items from the Italian version (4, 8, 16, 25, 32, 24, 26); thus, the scores ranged from 0 to 23 for SP and from 0 to 18 in SR, with higher scores reflecting, respectively, higher sensitivity to punishment and to reward.

#### 2.4.4. Food Neophobia Scale

The evaluation of food neophobia was quantified by the scale developed by [58], validated in Italian by Laureati et al. [59] This psychological trait describes the reluctance to eat and try new and unfamiliar products. It was investigated by means of 10 items, represented by 10 statements evaluated on a 7-point Likert scale (range: 1 = disagree strongly; 7 = agree strongly). The final score for the Food Neophobia Scale (FNS) was represented by the sum of ratings (ranging from 10 to 70).

#### 2.4.5. Sensitivity to Disgust

The individual sensitivity to core disgust was evaluated with the DS-SF questionnaire; this questionnaire is a short form of the Disgust Scale [60,61,62,63] validated in Italian by Spinelli et al [45], represented by 8 items divided into 2 subscales. Specifically, a 5-point scale was used, and in the case of Subscale 1, scores rated from 1 (strongly disagree-very untrue about me) to 5 (strongly agree-very true about me); rates for Subscales 2 ranged from 1 (not at all disgusting) to 5 (extremely disgusting). The total score for sensitivity to disgust was given by the sum of scores (ranging from 8 to 40).

### 2.5. Eating Behaviors, Food-Related Lifestyles, and Attitude Measurements

#### 2.5.1. Food-Related Lifestyle

The Food-Related Lifestyles (FRL) questionnaire [64], validated in Italian by Saba et al. [65], was used to measure the lifestyle, defined as the system of cognitive categories, scripts, and their associations, which associate a set of products to a set of values, related to food. The FRL is organized into 5 domains (and their relative 23 subscales) including ways of shopping (6 subscales), importance of quality aspects (6 subscales), cooking methods (6 subscales), consumption situations (2 subscales), and purchasing motives (3 subscales). The 69 items (7 of them required reverse scoring) were evaluated on a 7-point Likert scale (1 = disagree strongly; 7 = agree strongly). The total score for each subscale was calculated as the mean of scores given by respondents.

#### 2.5.2. Health and Taste Attitudes Scale

The individual orientation towards the hedonic and health characteristics of food was measured with the Health and Taste Attitudes Scale (HTAS) questionnaire [66] validated in Italian by Saba et al. [65], organized into 6 different domains: three domains were health-related (the general health interest (GHI), i.e., interest in eating healthily; light product interest (LPI), i.e., interest in eating reduced-fat foods; and natural product interest (NPI), i.e., the interest in eating foods that do not contain additives and are unprocessed), and three domains were taste-related (craving for sweet foods (CSF), food as a reward (FR), and pleasure (P)}). The HTAS included a total of 38 items, rated on a 7-point Likert scale (1 = disagree strongly; 7 = agree strongly). The HTAS for each domain was calculated as the mean of the ratings.

#### 2.5.3. Dutch Eating Behavior Questionnaire

The Dutch Eating Behavior Questionnaire (DEBQ) was used to measure individual differences in emotional eating (eating in response to internal emotional factors), external (eating in response to external factors such as the sight and smell of food), and restrained eating (eating less than desired to lose or maintain a particular body weight) [67], validated in Italian by Dakanalis et al. [68]. DEBQ consisted of 33 items rated on a 5-point scale (1 = never; 2 = seldom; 3 = sometimes; 4 = often, and 5 = very often). The total score for each domain was calculated as the mean.

### 2.6. Stated Liking, Familiarity with, and Choice of Meat-Based Products

#### 2.6.1. Familiarity with and Stated Liking of Meat-Based Products

Familiarity with and liking of meat-based fat-rich products were measured using a selection of the IT-Food Familiarity Questionnaire (IT-FFQ) and of the IT-Food Preference Questionnaire (IT-FPQ; [46], developed within the Italian Taste project to collect information about familiarity with and liking of foods among Italians. The IT-FFQ and IT-FPQ included 15 items referred to meat, part of a larger group of 184 items. IT-FFQ was assessed using a 5-point labelled scale (1 = I do not recognize it; 2 = I recognize it, but I have never tasted it; 3 = I have tasted it, but I do not eat it; 4 = I occasionally eat it; 5 = I regularly eat it; [69]). Stated liking of meat-based fat-rich products was assessed using a 9-point hedonic labelled scale [70] with the addition of the option “never tasted it”. The presentation order of the items in both questionnaires was randomized across participants.

Meat products were classified as fat and low-fat based on macro-composition. Fat products were further divided into meat and cold meat based on processing (raw and cured). Three groups were then obtained: fat-rich meat, fatty cold meat, and low-fat meat items (Table 2). Indices of familiarity and liking were calculated for each of the three as the sum (for familiarity) and the mean (for liking) of the individual scores of each subject.

#### 2.6.2. Choice of Fat-Rich/Not Fat-Rich Meat-Based Foods and Validation of the Oppositions

Choice of fat-rich/not fat-rich meat-based foods was quantified using a selection of items taken from the IT-Food Choice Questionnaire [46], a tool developed in order to evaluate preference within a pair of foods selected among the ones included in the IT-FFQ/FPQ. For each pair, respondents were asked to indicate which meat they would choose in a main meal (either lunch or dinner). The presentation order of the food items within each pair and of the pairs was randomized across participants. A choice index for fat-rich meat (FCI) was calculated as the sum of the choices of the fattier and more caloric option assigning to each a value of 1, with higher scores reflecting a greater choice of the fattier options.

The options were selected based on a preliminary study conducted at the University of Florence with 181 subjects (mean age 40.5, range 18–68; 74.86% women) using a check-all-that-apply methodology to evaluate sensory properties (only results about “fatty” and “caloric” will be presented here) though an online questionnaire. Products were presented monadically in a randomized order. Attributes were presented in a randomized order.

### 2.7. Data Analysis

The Cochran’s Q test was applied to check for significant differences among products in the preliminary study for the choice questionnaire validation.

Cronbach’s α was calculated for all questionnaires. The effect of gender, age, and gender * age was calculated on all the variables (PROP status, psychological traits, attitudes, stated liking of and familiarity with fatty meat, cold meat, and low-fat) using a two-way ANOVA. The Chi-squared test was applied to check the difference in the distribution of PROP status by gender.

A three-way ANOVA model was applied to test the effect of PROP, age, gender and their intercation on liking of and familiarity with fatty meat, cold meat, and low-fat meat.

Indices of liking, familiarity with, and choice of fat-rich meat were calculated, respectively, as the mean (for liking) and the sum (for familiarity and choice) of the ratings.

Individuals higher and lower in fat-rich meat choice were identified, respectively, as those higher (high FCI) and lower (low FCI) than the median of the choice index. Subjects whose values corresponded to the median were not considered. One-way ANOVA models were applied to test the effect of choice for fat-rich meat (high/low) on each variable considered in the study by gender. The significance level was set at 0.05, but results <0.1 are discussed, for the exploratory purposes of the study.

A partial least squares discriminant analysis (PLS-DA) regression model was computed on the whole dataset and separately for each gender assuming a low and high fat choice index as the response variable (Y) and 39 explanatory variables (X): age; seven personality traits (FN, SR, SP, DS, and TAS subscales, see Table 3); PROP responsiveness; attitudes (selected subscales reported in Table 4); familiarity with and liking of fat-rich meat, cold meat, and low-fat meat.

All PLS regression models were run on standardized mean centered input variables, using cross-validation on 5 (for men) and 6 (for women) random segments and performing a jack-knife uncertainty test with a 95% confidence interval for the detection of significant variables [71] (Due to the large amount of information collected, a two-step procedure was used [72]. In the first step, all the individual attributes were included in the model. Then, in further steps, a new model was run only including as active variables those that were found to be significant in the model according to the uncertainty test. The other variables were included in the model as downweighted. This resulted in a better suited and more parsimonious model.

All data were analyzed using XLSTAT 19.4.1, with the exception of the PLS-DA, which was conducted using Unscrambler^®^X 10.3.

## 3. Results

### 3.1. PROP Responsiveness by Gender and Age

Men and women significantly differed in PROP responsiveness (*Χ*^2^ = 32.48, *p* < 0.0001). The distribution of men into NT, MT, and ST reflected quite well the expected distribution of 25%, 50%, and 25% respectively, while in women, a higher number of supertasters was found (41.28%). PROP responsiveness decreased with age (F = 13.60, *p* < 0.0001), with an interaction with gender close to significance (*p =* 0.052).

### 3.2. Psychological and Personality Traits by Gender and Age

The internal reliability of all the questionnaires measuring psychological traits was satisfactory (Table 3). An interaction between gender and age was found in the case of private body consciousness (F = 7.85; *p =* 0.0004) and sensitivity to disgust (F = 3.17; *p =* 0.042), with women higher in these traits than men independent of age, with the exception of individuals aged 18 to 30, for which the two genders did not differ in PBC. Women were found also to have more difficulty in identifying the feeling dimension (alexithymia-DIF) and less difficulty in externally oriented thinking (alexithymia-EOT), a higher private body consciousness, a higher sensitivity to punishment, and a lower sensitivity to reward than men.

Alexithymia and its subscales DIF, DDF, and EOT, and sensitivity to punishment and to reward were higher in the younger group. Inversely, neophobia and sensitivity to disgust increased with age.

### 3.3. Eating Behaviors, Food-Related Lifestyles, and Attitude Towards Health and Taste by Gender and Age

Nine (out of the twenty-three) subscales of the food-related lifestyle scale (APA2—price/quality relation, APA3—novelty, APA5—taste, CS4—whole family, CS5—planning, US1—snacks versus meal, US2—social event, CO2—security, CO3—social relationship) and the pleasure domain of the Health and Taste Attitude Scale showed a low internal validity (α < 0.6) and were removed from the analysis. Age and gender effects were found on some subscales, without a significant interaction between these two variables with only one exception (Table 4): the belief that cooking was a woman’s task (CS6) was higher in women and in men aged 45–60. Women found the price criteria in food purchasing (SC5) and the use of a shopping list (SC6) more important than men. Women were more interested in looking for new ways of cooking (CS2) and declared more self-fulfillment with food (CO1), while men declared using more convenience foods than women (CS3). No difference was found between genders in the importance of product information (SC1), attitudes toward advertising (SC2), enjoyment from food shopping (SC3), interest in specialty shops (SC4), interest in organic products (APA4), importance of product freshness (APA6), and interest in cooking (CS1).

Enjoyment from food shopping (SC3), the importance of the price criteria (SC5), and interest in cooking decreased with age (CS1), while the interest in specialty shops (SC4) and organic products (APA4) increased with age. The consumption of convenience foods was lower among the 30–35-year-olds, but with older age, this tended to increase (CS3).

Health interest was higher for women than men; this was confirmed both by the FRL Health subscale (APA1) and by the HTAS (GHI). Health interest also increased with age. Women were also more interested in the naturalness of the products (namely, absence of additives), and they had a greater attitude for craving sweet foods. Interest in light products was higher among the 18–30 age class, while the interest in natural products increased with age. The younger used more food as a reward than older individuals.

Women had a greater attitude toward emotional eating with no age effect. External eating did not differ by gender and decreased with age. A significant interaction between gender and age was found for restrained eating, which was higher for women and increased with age only in men (F = 5.06; *p =* 0.006).

### 3.4. Liking of, Familiarity with, and Choice of Meat-Based Products

#### 3.4.1. Stated Liking of and Familiarity with Meat-Based Products

The percentage of individuals who had never tasted meat products was generally low, and lower in men than women. Lamb and carpaccio were the two products with the highest percentage of individuals who declared to have never tasted the product; 6.67% of women and 3.58% of men in the case of lamb; 8.51% of women and 4.77% of men for carpaccio. Only 3.31% of the participants declared a vegetarian diet (3.83% among women and 2.58% among men).

In general, all the products were liked with average ratings ranging from 6.70 to 7.88 for men and 5.43 to 7.78 for women. Men stated a liking higher than women for lamb, pork, beef rib, steak, carpaccio, grilled beef cutlet, and pork sausage, while no difference between genders was found for breaded cutlet and chicken breast. A similar trend was found also in fatty cold meat products with men reporting a higher liking of mortadella, spicy salami, and salami. No gender difference was found for ham (cooked or dry-cured) and bacon.

Indices of familiarity with and liking of meat were calculated. Internal reliability, gender, and age effects are reported in Table 5. Internal reliability was acceptable, with Cronbach’s α ranging from 0.60 to 0.84. Gender affected the liking of and familiarity with fatty meat and fatty cold meat indices and the liking of low-fat meat index, with women reporting a lower liking and familiarity index than men. Liking of meat independently of fat and familiarity with fatty cold meat and low-fat meat decreased with age. The familiarity with fatty meat index remained stable with age. No interaction between gender and age was observed.

#### 3.4.2. Stated Liking of and Familiarity with Meat-Based Products by PROP Status

**Figure 2:**
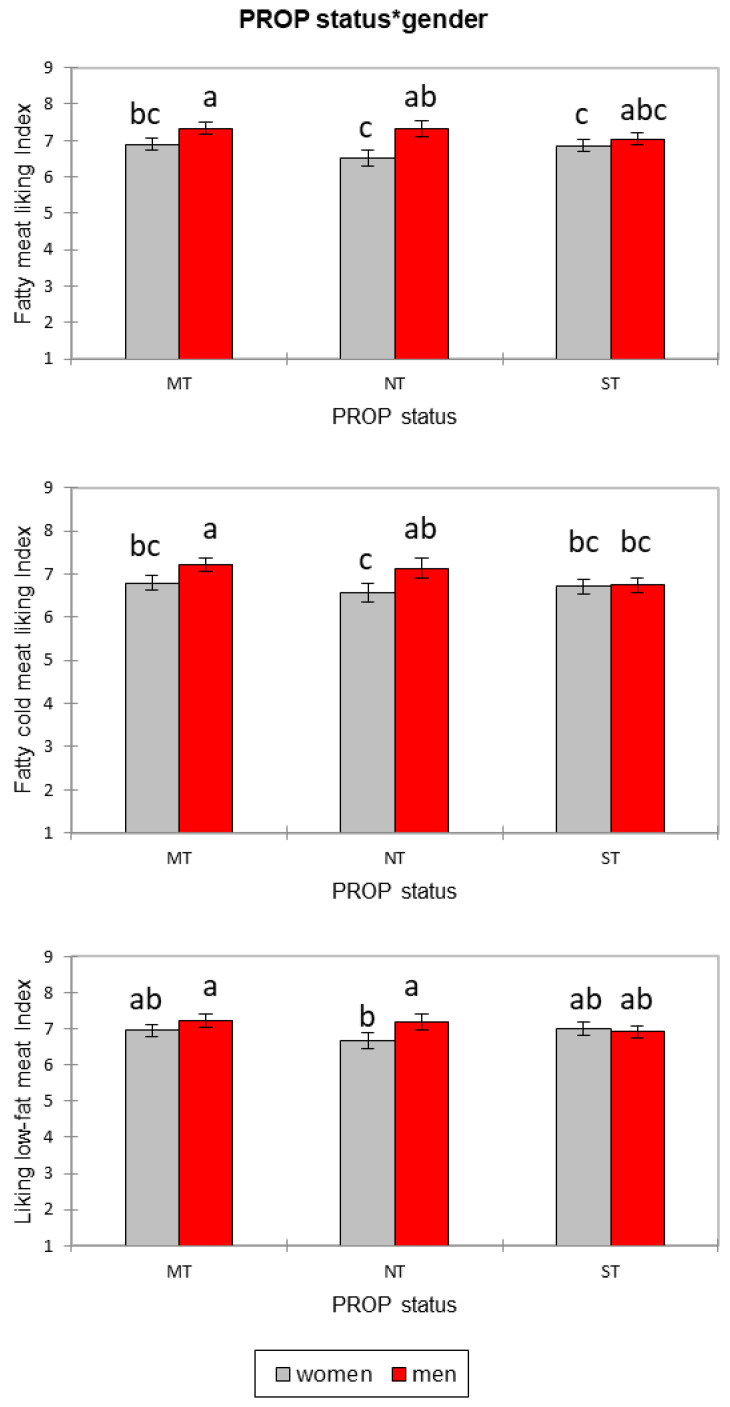
Differences by gender and PROP status (MT = medium taster; NT = non-tasters; ST = supertasters) in mean liking of fatty meat, cold meat, and low-fat meat.

PROP status was found to interact with gender with a significant effect on liking of fatty meat (F = 3.36, *p* = 0.035), liking of cold meat (F = 3.09, *p* = 0.046), liking of low-fat meat (F = 3.32, *p* = 0.036), and familiarity with low-fat meat (F = 3.17, *p* = 0.042). Post hoc tests showed that men declared a higher liking of fat and cold meat compared to women and that medium and non-taster men expressed a higher liking compared to medium and non-taster women, respectively. No gender difference in liking fat and cold meat was found in supertasters. Men and women did not differ in liking of low-fat meat, with the exception of non-tasters: female non-tasters declared a lower liking than male non-tasters (Figure 2). A further interaction between age, gender, and PROP status was found on liking of fatty meat (F = 3.35, *p* = 0.010): results showed that liking for fatty meat was lower in women aged 30–45 non-tasters and in men aged 45–60 supertasters. A post hoc test did not indicate significant difference in the case of familiarity with low-fat meat.

#### 3.4.3. Fat-Rich Meat Choice Index

Preliminary Study: Validation of the Fat-Rich Meat Choice Index: Cochran’s Q test applied on each attribute showed a significant difference between the two options of each choice pair both in fatty and in caloric (Table 6).

Correlation analysis showed that the choice in the pair 2 (grilled vs. breaded cutlet) and 6 (carpaccio vs. sliced steak) was not significantly correlated with the choice in other pairs, and factor loading was lower than 0.3 for these pairs. Internal reliability improved upon removing these items with Cronbach’s α passing from 0.58 to 0.68; thus, only five pairs (bold in Table 7) were retained to calculate the fat-rich meat choice index.

Factors Affecting Fat-Rich Meat Choice by Gender: An effect on the fact-rich meat choice index of gender (F = 62.11, *p* < 0.0001) and age (F = 6.86, *p =* 0.001) not further characterized by interaction was found. The fat-rich meat choice index was lower in women and in individuals aged 18–30. Given the higher variability in choice by gender, analyses were conducted separated in women and men.

Individuals low and high in the fatty meat choice index differed mainly in familiarity with and liking of fat-rich meat and fatty cold meat, both in men and women (Table 7). In addition, individuals low in fat choice declared a higher general health interest, a lower susceptibility to external eating, higher neophobia, and tended to be younger in both genders. Some variables affected choice only within one gender: women reporting a lower fat-rich meat choice index resulted in being more sensitive to disgust and to punishment, less sensitive to emotional eating, less self-fulfilled in food, and enjoyed food shopping less. On the contrary, men who reported a lower fat-rich meat choice index gave a higher importance to product information and to organic products, declared a higher light product interest, and resulted in being less prone to use food as a reward.

The partial least squares discriminant analysis regression on the whole sample showed that gender was the most relevant variable impacting choice of meat low and high in fat. Gender was found to affect significantly the choice of fat-rich meat, with men choosing systematically the fattiest option compared to women. For this reason, we proceeded with an analysis separated by gender.

In both models, the cross-validation indicated that three factors had a significant prediction ability and were used in the jack-knife test for estimating the uncertainty of the model parameters.

**Figure 3:**
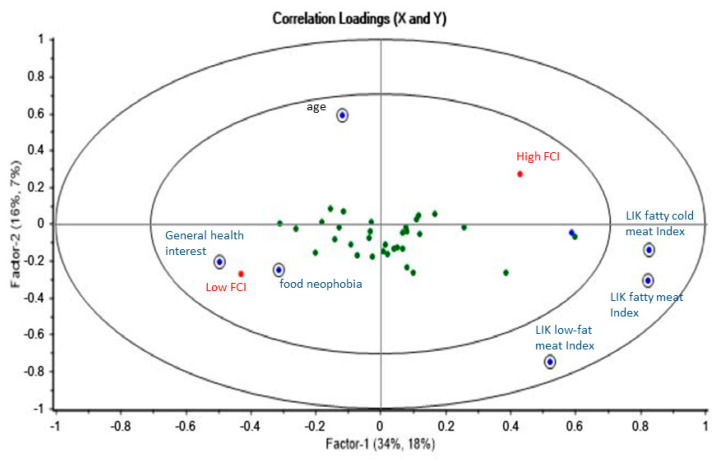
Correlation loadings with significant consumer attributes from the PLS-DA model in men. Significant variables according to the uncertainty test are circled.

**Figure 4:**
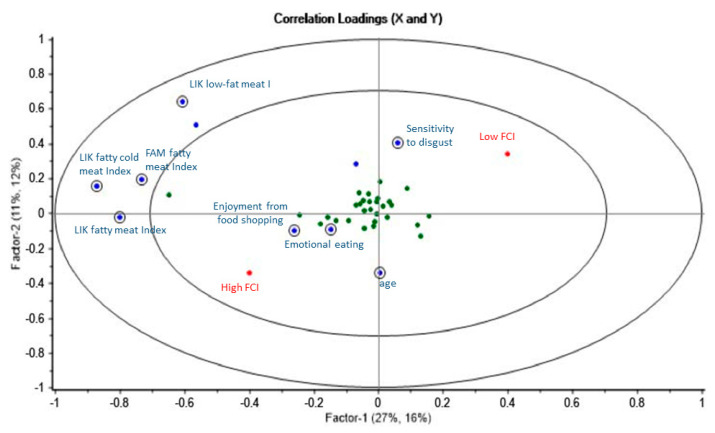
Correlation loadings with significant consumer attributes from the PLS-DA model in women. Significant variables according to the uncertainty test are circled.

The explained variance for the first three components was 34%, 16%, and 5% for X and 18%, 7%, and 2% for Y in men (Figure 3) and 27%, 11%, and 6% for X and 16%, 12%, and 2% for Y in women (Figure 4).

In men (*n* = 393), on the first component, the liking of cold meat was positively associated with the choice of fatty meat, while general health interest and neophobia were negatively correlated with it. On the second and third component, also age and the liking of fatty meat were positively associated with fatty meat choice, while liking of low-fat meat was negatively associated with it (Figure 3).

In women (*n* = 522), on the first component, liking fat-rich meat and cold meat and familiarity with fat-rich meat, enjoyment from food shopping (SC3), and emotional eating were positively associated with a higher fat choice index, while sensitivity to disgust was negatively associated with it. On the second component, liking low-fat meat was negatively associated with the fat choice index, while age was positively associated with it (Figure 4).

## 4. Discussion

The gender-sensitive approach adopted in this study allowed analyzing differences and similarities between and across women and men. First, through a between-gender approach, the effect of gender was investigated together with age, both on the variables characterizing the subjects (personality traits, attitudes, and taste responsiveness) and on liking of, familiarity with, and choice of meat varying in fat. Secondly, a within-gender approach was adopted. This allowed pointing out similarities and differences in women and men, respectively, that remain often underestimated as they are covered by the larger differences between genders. The advantage of this latter approach is that it provides information that can be used to develop gender-specific interventions to promote healthier food behaviors.

Gender was found to have a major impact on liking of and familiarity with meat and choice of fat-rich meat. A lower liking of meat in general was found in women, independent of fat content. Women also reported a lower familiarity than men with fatty meat and cold meat. These results were in line with previous studies that reported a lower intake of meat in women [15,73]. In addition, this study showed that this was true independent of fat content. Looking at behaviors, even if women expressed a lower liking also for low-fat meat compared to men, in terms of consumption, they did not differ from men, with a low-fat meat familiarity index that was low in both genders. This suggested that men, even if they liked all types of meat, tended to consume less meat lower in fat, such as chicken breast. In the choice between a meat option lower or higher in fat, women systematically chose the option that was lower in fat.

Age was differently associated with liking of, familiarity with, and choice of fat-rich meat. In fact, while younger individuals declared a higher liking of meat higher in fat, when they had to choose between two options, higher and lower in fat, they preferred the option lower in fat. These results suggested that in older individuals, there was a higher coherence between liking, consumption, and choice, while in younger individuals, the conflict between liking and choice was greater. In general, younger individuals liked all meat more independently of its fat content and were more familiar with cold and low-fat meat, but when they could freely choose, they chose more consistently the option lower in fat. Older individuals reported a lower liking of all meat independently of fat and did not differ from the younger in familiarity with fat-rich meat; however, when they could freely choose, they systematically chose the option richer in fat. This meant that for these subjects, consuming the option lower in fat was more difficult, notwithstanding that for them, adopting healthier food behaviors was even more relevant and recommended.

Univariate analysis showed that many, but not all the variables that were found to affect choice of meat higher in fat significantly played a role in both genders. Liking of and familiarity with meat and cold meat higher in fat were positively associated with choice of meat higher in fat, as could be expected, in both genders. Health interest was found to be higher in women, in line with previous findings [73], and was associated in both genders with a lower choice of fat-rich meat. Neophobia was negatively associated with fat-rich meat choice both in men and women. Several studies reported neophobics to have a lower diet variety, lower liking [74], and lower intake [75,76] of meat and fish. Furthermore, fattier meat such as lamb may be less familiar, namely more novel, and thus less appealing for neophobics. We may also hypothesize that this result is related to an association of fat with more flavorful options, while neophobics usually prefer milder tastes [59].

Different from previous studies, we did not find any association of restrained eating with fat-rich meat choice, while we found in both men and women a positive association of external eating, defined as eating in response to external food-related cues, such as the sight and smell of food, regardless of physical need, in line with [38]. The lack of a relationship with restrained eating may be explained by the fact that in our study, the range of age was wider compared to [37], and individuals were not selected for specific health conditions; in addition in the choice task, individuals were encouraged to freely choose as they were in a condition without restriction (“if you are on a diet, please answer as if you are not on a diet”).

In women, we also found a positive association with emotional eating, which suggested that choice of meat rich in fat was higher in those women that eat more in response to negative emotions. This may be explained by the nutritional and rewarding value of fat that helps to cope with stress [25,77,78].

Sensitivity to disgust and sensitivity to punishment as a tendency were associated negatively, only in women, with higher fat-rich meat choice. We may hypothesize that the impact of sensitivity to disgust was related to visceral aspects of how the product looks: it is common that meat, especially when fat is visible, is perceived as more disgusting, particularly by those individuals more sensitive to this. Sensitivity to punishment, compared to sensitivity to disgust, seemed to be more related to the value that was associated with the food more than with how the food looked like. This trait refers, in fact, to the degree to which an individual’s behavior is inhibited by punishment-relevant stimuli and was found to be associated with food avoidance [79]. We may hypothesize that choice for higher fatty meats was associated with punishment, as an unhealthier behavior. Individuals more sensitive to punishment might choose more systematically the lower fat option to avoid punishment, as a sort of protective and prudent behavior.

We did not find an association between sensitivity to reward and higher fat-rich meat choice, while we found an association with the attitude to use food as a reward only in men. Sensitivity to reward refers to the degree to which an individual’s behavior is motivated by reward-relevant stimuli. Previous studies [39,40] that highlighted the association between sensitivity to reward and fat liking focused mainly on fat in sweet foods. Our results showed that further studies are required to extend these considerations to fat in salty foods, and particularly in meat.

Although current results about PROP responsiveness and fat perception are controversial [27], several studies found that PROP status was negatively associated with preference for dietary fat in women and girls [80,81] or independently of the gender [29,30,82,83]. We did not observe any difference according to PROP status in liking of, familiarity with, or choice of fat-rich meat, neither in women nor in men. When we found an effect, on liking, gender moderated this relationship. An interaction between PROP responsiveness and personality has been reported [84,85]. This may suggest that PROP status is interlinked with personality traits, thus moderating its effect. Another possible explanation for this result is the specific food product considered, meat, which is more liked by men. In this food matrix, fat content could be less relevant compared to the specificity of the food matrix (meat).

In men, a lower choice of fat-rich meat was associated also with a higher attitude toward light products, organic food, and importance assigned to product information. This suggested that cognitive motivations, that is knowledge about product nutritional and health characteristics, were very relevant for this gender in choosing the option lower in fat, compared to women.

The multivariate models separated by gender allowed pointing out the main factors that acted as barriers or facilitators of the consumption of fat-rich meat. In both genders, liking of and familiarity with meat confirmed their relevant role in choice. Liking of fat-rich meat and cold meat was positively associated with higher fat-rich meat choice, while familiarity with low-fat meat was negatively associated with it. Apart from liking of and familiarity with meat, women and men differed substantially in the main factors that affected fat-rich meat choice. Women were in general more interested in health than men, and this may explain the main difference in choice of fat-rich meat between genders. However, when we look at each gender separately, general health interest was significantly correlated with a lower choice of fat-rich meat only in men. This difference may be explained by the fact that women shared their interest in health, and thus, this factor did not play a significant role in choosing the option lower in fat. In men, factors that discouraged the choice of fat-rich meat seemed mainly represented by a higher general health interest and food neophobia. This meant that for men, cognitive motivations were very relevant as a deterrent for choosing the fat-rich option, both in terms of importance attributed to the information available to the individual (e.g., in terms of health benefits) and in terms of past experience, which they referred to when trying to make sense of information presently available and in determining how to respond or relate to the current situation. This latter aspect is very relevant to neophobia, which is defined as reluctance to eat unfamiliar foods.

On the contrary, in women, greater fat-rich meat choice was found to be inversely related to sensitivity to disgust and positively to emotional eating and enjoyment of food shopping and, thus, more an aspect of emotions than of attitudes towards health.

The large sample of individuals that participated in this study warranted the reliability of the results. However, the study was conducted on self-reported preferences on food items presented using words. The extension of the approach to tasted food differing in fat content could be helpful to confirm these results.

## 5. Conclusions

The study showed that women like meat less than men and chose the option lower in fat more compared to men. In addition, thanks to the large sample and the gender-sensitive approach adopted, this study showed that different factors affected the choice of fat-rich meat by gender, in addition to liking of and familiarity with fat-rich and cold meat and age. These findings have important health implications. They suggest that strategies personalized by gender to reinforce or activate barriers to the choice of fat-rich meat may be more effective to reduce fat intake, promoting the consumption of meat lower in fat. Men were found to be more responsive to cognitive motivations, such as health benefits, neophobia, and information about the product. This type of motivation did not seem to be very effective for women, who were in general more interested in health independently of the choice of fat-rich meat. Barriers to choose meats richer in fat in women were more psychological, related to sensitivity to disgust and punishment, which may lead to inhibitory behaviors, while emotional eating may act as a facilitator.

## Figures and Tables

**Table 1:** Details of the research participants.

Characteristics of the Participants	%
Gender (women)	58.36
Age range	%
18–30	45.6
31–45	27.8
46–60	26.6
Education level	%
none	0.08
elementary school	0.33
middle school	4.39
high school	49.25
degree	32.17
post degree	13.76
Marital status	%
not married	58.69
married	35.79
divorced	4.50
widowed	1.02
Family members (*n*)	3.37 (1.27 SD)
Expense for food (monthly, €)	%
up to 200	18.82
from 201 to 400	43.78
from 400 to 600	28.77
more than 600	8.62
Diet	%
none	91.13
hypocaloric diet	6.52
specific diet for health reasons	2.35

**Table 2:** List of meat-based products selected for the fatty meat, fatty cold meat, low-fat meat stated liking and familiarity indices.

Stated Liking/Familiarity		
Fatty Meat	Fatty Cold Meat	Low-Fat Meat
Beef rib	Bacon	Carpaccio
Breaded cutlet	Cooked ham	Chicken breast
Lamb	Cured ham	Grilled cutlet
Pork	Mortadella	
Pork sausage	Salami	
Steak	Spicy salami	

**Table 3:** Effect of gender (women and men) and age (age classes: 18–30, 31–45, 46–60) on personality traits. For each trait, Cronbach’s α, mean scores by gender and age class, and F- and *p*-values are reported. SF, Short Form.

Personality Trait	Cronbach’s α	Gender				Age				
		Women	Men	F	*p*	18–30	31–45	46–60	F	*p*
Toronto Alexithymia Scale (TAS-20)	0.82	46.21	45.98	0.12	0.734	**49.75 ^a^**	**43.44 ^b^**	**45.09 ^b^**	37.11	**<0.0001**
Identifying feelings dimension (DIF)	0.82	**15.35 ^a^**	**14.49 ^b^**	6.7	**0.010**	**16.59 ^a^**	**13.23 ^c^**	**14.94 ^b^**	37.86	**<0.0001**
Describing feelings dimension (DDF)	0.78	12.67	12.94	0.94	0.333	**14.06 ^a^**	**12.16 ^b^**	**12.20 ^b^**	23.97	**<0.0001**
Externally oriented thinking (EOT)	0.61	**17.96 ^b^**	**18.78 ^a^**	8.33	**0.004**	**19.11 ^a^**	**18.05 ^b^**	**17.94 ^b^**	8.08	**0.000**
Private Body Consciousness (PBC) *	0.72	**18.68 ^a^**	**17.36 ^b^**	33.69	**<0.0001**	18.22	18.11	17.74	1.63	0.197
Sensitivity to punishment (SP)	0.91	**9.86 ^a^**	**8.05 ^b^**	36.54	**<0.0001**	**10.52 ^a^**	**8.15 ^b^**	**8.20 ^b^**	32.04	**<0.0001**
Sensitivity to reward (SR)	0.87	**5.13 ^b^**	**6.84 ^a^**	74.03	**<0.0001**	**7.64 ^a^**	**5.57 ^b^**	**4.75 ^b^**	85.9	**<0.0001**
Food neophobia (FN)	0.86	27.24	27.65	0.36	0.548	**26.08 ^b^**	**26.64 ^b^**	**29.62 ^a^**	9.92	**<0.0001**
Sensitivity to disgust (SD-SF) *	0.70	**30.63 ^a^**	**27.56 ^b^**	90.42	**<0.0001**	**28.04 ^b^**	**29.22 ^a^**	**30.03 ^a^**	14.26	**<0.0001**

Significant differences are in bold. ^a,b,c^ Different letters indicate a significant difference (*p* < 0.05).

* Variable for which a significant interaction (*p* < 0.05) between gender and age was found.

**Table 4:** Effect of gender (women, men) and age (age classes: 18–30, 31–45, 46–60) on attitudes toward foods. For each subscale, Cronbach’s α, mean scores by gender and age class, and F- and *p*-values are reported. Only subscales with Cronbach’s α > 0.6 α are reported.

**Food-Related Lifestyle (FRL) Questionnaire**											
**Subscale**		**Cronbach’s α**	**Gender**				**Age**				
			**Women**	**Men**	**F**	***p***	**18–30**	**31–45**	**46–60**	**F**	***p***
Way of shopping											
SC1	Importance of product information	0.74	5.49	5.29	0.29	0.746	5.30	5.42	5.46	0.08	0.972
SC2	Attitudes toward advertising	0.60	3.05	3.05	0.01	0.986	3.12	3	3.02	0.03	0.993
SC3	Enjoyment from food shopping	0.64	5.54	5.47	0.92	0.337	**5.59 ^a^**	**5.61 ^a^**	**5.32 ^b^**	6.36	**0.002**
SC4	Specialty shops	0.74	4.55	4.56	0.01	0.926	**4.37 ^b^**	**4.51 ^b^**	**4.79 ^a^**	9.12	**0.000**
SC5	Price criteria	0.69	**4.83 ^a^**	**4.62 ^b^**	6.69	**0.010**	**4.91 ^a^**	**4.65 ^b^**	**4.61 ^b^**	5.95	**0.003**
SC6	Shopping list	0.70	**4.98 ^a^**	**4.73 ^b^**	9.67	**0.002**	4.77	4.93	4.88	1.63	0.196
Quality aspects											
APA1	Health	0.82	**5.68 ^a^**	**5.51 ^b^**	6.05	**0.014**	**5.26 ^c^**	**5.59 ^b^**	**5.94 ^a^**	32.11	**<0.0001**
APA4	Organic product	0.77	4.52	4.47	0.33	0.568	**4.20 ^b^**	**4.38 ^b^**	**4.91 ^a^**	25.93	**<0.0001**
APA6	Freshness	0.75	6.26	6.19	1.81	0.179	6.17	6.24	6.27	1.44	0.238
Cooking methods											
CS1	Interest in cooking	0.78	5.41	5.36	0.4	0.547	**5.44 ^a^**	**5.53 ^a^**	**5.19 ^b^**	5.34	**0.005**
CS2	Looking for new ways	0.78	**5.43 ^a^**	**5.24 ^b^**	7.0	**0.008**	5.42	5.37	5.21	2.71	0.067
CS3	Convenience	0.71	**2.35 ^b^**	**2.67 ^a^**	19.1	**<0.0001**	**2.62 ^a^**	**2.38 ^b^**	**2.53 ^a,b^**	4.17	**0.016**
CS6	Woman’s task *	0.64	**2.40 ^a^**	**1.98 ^b^**	33.72	**<0.0001**	**2.07 ^b^**	**2.11 ^b^**	**2.39 ^a^**	7.5	**0.001**
Purchasing motives											
CO1	Self-fulfilment in food	0.67	**5.44 ^a^**	**5.21 ^b^**	10.92	**0.001**	5.35	5.32	5.30	0.25	0.781
**Health and Taste Attitudes Scale (HTAS)**											
**Domain**		**Cronbach’s α**	**Gender**				**Age**				
			**Women**	**Men**	**F**	***p***	**18–30**	**31–45**	**46–60**	**F**	***p***
General health interest		0.78	**4.90 ^a^**	**4.64 ^b^**	20.04	**<0.0001**	**4.52 ^c^**	**4.72 ^b^**	**5.07 ^a^**	31.87	**<0.0001**
Light product interest		0.81	3.40	3.42	0.10	0.753	**3.61 ^a^**	**3.41 ^b^**	**3.21 ^b^**	12.26	**<0.0001**
Natural product interest		0.74	**4.62 ^a^**	**4.42 ^b^**	9.09	**0.003**	**4.15 ^c^**	**4.46 ^b^**	**4.96 ^a^**	54.59	**<0.0001**
Craving for sweet foods		0.85	**5.09 ^a^**	**4.37 ^b^**	73.43	**<0.0001**	4.87	4.66	4.67	3.07	**0.047**
Using food as a reward		0.81	4.49	4.39	1.68	0.195	**4.68 ^a^**	**4.54 ^a^**	**4.11 ^b^**	20.87	**<0.0001**
**Dutch Eating Behavior Questionnaire (DEBQ)**											
**Domain**		**Cronbach’s α**	**Gender**				**Age**				
			**Women**	**Men**	**F**	***p***	**18–30**	**31–45**	**46–60**	**F**	***p***
Restrained eating *		0.88	2.95	2.68	1.54	0.216	**2.73 ^b^**	**2.74 ^b^**	**2.98 ^a^**	5.25	**0.001**
Emotional eating		0.94	**2.44 ^a^**	**1.99 ^b^**	4.21	**0.015**	2.43	2.15	2.06	0.88	0.45
External eating		0.82	3.19	3.21	0.04	0.961	**3.39 ^a^**	**3.20 ^b^**	**3.02 ^c^**	5.49	**0.001**

Significant differences are in bold. ^a,b,c^ Different letters indicate a significant difference (*p* < 0.05). * Variable for which a significant interaction (*p* < 0.05) between gender and age was found.

**Table 5:** Effect of gender (women, men) and age (age classes: 18–30, 31–45, 46–60) on the familiarity with (FAM) and liking (LIK) of fatty meat, fatty cold meat, and low-fat meat indices. For each index, Cronbach’s α, mean scores by gender and age class, and F- and *p*-values are reported.

Indices	Cronbach’s α	Gender				Age				
		Women	Men	F	*p*	18–30	31–45	46–60	F	*p*
FAM fatty meat (range: 1–30)	0.82	**23.32 ^b^**	**23.97 ^a^**	11.34	**0.001**	23.72	23.88	23.33	2.57	0.077
FAM fatty cold meat (range: 1–30)	0.82	**23.72 ^b^**	**24.23 ^a^**	7.57	**0.006**	**24.21 ^a^**	**24.12 ^a,b^**	**23.59 ^b^**	4.31	**0.014**
FAM low-fat meat (range: 1–15)	0.60	12.22	12.24	0.03	0.868	**12.28 ^a^**	**12.42 ^a^**	**11.99 ^b^**	5.39	**0.005**
LIK fatty meat (range: 1–9)	0.84	**6.77 ^b^**	**7.29 ^a^**	36.91	**<0.0001**	**7.23 ^a^**	**7.07 ^a^**	**6.79 ^b^**	9.74	**<0.0001**
LIK fatty cold meat (range: 1–9)	0.81	**6.70 ^b^**	**7.10 ^a^**	23.31	**<0.0001**	**6.97 ^a^**	**7.07 ^a^**	**6.67 ^b^**	7.26	**0.001**
LIK low-fat meat (range: 1–9)	0.69	**6.90 ^b^**	**7.16 ^a^**	9.59	**0.002**	**7.26 ^a^**	**7.12 ^a^**	**6.71 ^b^**	15.56	**<0.0001**

Significant differences are in bold. ^a,b^ Different letters indicate a significant difference (*p* < 0.05).

**Table 6:** Proportion of subjects who checked fat and caloric to describe the low- and high-fat option between each pair. The amount of lipids for 100 g is reported (source: food composition tables; CREA—Centro di Ricerca per gli Alimenti e la Nutrizione, Ministero per le Politiche Agricole, Alimentari e Forestali, http://sapermangiare.mobi/tabelle_alimenti.html). Items selected for the fat-rich meat index are in bold.

	Choice		Fat			Caloric			Lipids (g/100 g)	
	Low-Fat (0)	High Fat (1)	*p-*Value	0	1	*p-*Value	0	1		
1	Calf rib	Lamb rib	0.000	0.436 ^a^	0.646 ^b^	0.000	0.470 ^a^	0.624 ^b^	6.1	4.2
2	Grilled cutlet	Breaded cutlet	0.000	0.182 ^a^	0.840 ^b^	0.000	0.271 ^a^	0.956 ^b^	-	-
3	Chicken breast	Sausage	0.000	0.083 ^a^	0.950 ^b^	0.000	0.105 ^a^	0.978 ^b^	0.9	26.1
4	Chicken	Lamb	<0.0001	0.083 ^a^	0.646 ^b^	<0.0001	0.105 ^a^	0.624 ^b^	10 *	14.2
5	Cooked ham	Mortadella	0.000	0.442 ^a^	0.923 ^b^	0.000	0.436 ^a^	0.912 ^b^	14.7	28.1
6	Carpaccio	Sliced steak (tagliata)	0.000	0.160 ^a^	0.354 ^b^	0.000	0.260 ^a^	0.475 ^b^	2.7	6.1
7	Cooked ham	Cured ham	0.005	0.442 ^a^	0.564 ^b^	0.024	0.436 ^a^	0.536 ^b^	14.7	23

* Without skin. ^a, b^ Different letters indicate a significant difference (*p* < 0.05).

**Table 7:** Effect of fatty meat choice index (low FCI, high FCI) on age, personality traits, and attitudes toward food by gender. F, *p*, and mean scores. Only variables with *p* < 0.1 are reported.

	Women				Men			
Variables	F	*p*	Low FCI	High FCI	F	*p*	Low FCI	High FCI
FAM fatty meat index	33.20	<0.0001	22.45 ^b^	24.03 ^a^	20.32	<0.0001	22.87 ^b^	24.45 ^a^
FAM fatty cold meat index	25.05	<0.0001	23.05 ^b^	24.34 ^a^	23.61	<0.0001	23.01 ^b^	24.71 ^a^
LIK fatty meat index	67.72	<0.0001	6.28 ^b^	7.33 ^a^	46.09	<0.0001	6.73 ^b^	7.60 ^a^
LIK fatty cold meat index	50.04	<0.0001	6.24 ^b^	7.10 ^a^	39.84	<0.0001	6.52 ^b^	7.36 ^a^
Age	3.61	0.058	34.04	36.20	3.17	0.076	34.12	36.67
Food neophobia	7.70	0.006	28.08 ^a^	25.30 ^b^	12.18	0.001	30.25 ^a^	25.90 ^b^
Sensitivity to disgust	6.27	0.013	31.09 ^a^	29.96 ^b^	-	-	-	-
Sensitivity to punishment	2.88	0.090	10.57	9.79	-	-	-	-
SC1 (importance of product information)	-	-	-	-	10.75	0.001	5.56 ^a^	5.08 ^b^
SC3 (enjoyment from food shopping)	8.60	0.004	5.41 ^b^	5.73 ^a^	-	-	-	-
APA4 (organic products)	-	-	-	-	3.23	0.073	4.58	4.30
CO1 (self-fulfillment in food)	5.37	0.021	5.32 ^b^	5.55 ^a^	-	-	-	-
Emotional eating	3.60	0.058	2.39	2.53	-	-	-	-
External eating	6.70	0.010	3.16 ^b^	3.29 ^a^	6.66	0.010	3.13 ^b^	3.31 ^a^
General health interest	6.92	0.009	5.02 ^a^	4.79 ^b^	25.42	<0.0001	4.96 ^a^	4.39 ^b^
Light product interest	-	-	-	-	6.78	0.010	3.68 ^a^	3.35 ^b^
Using food as a reward	-	-	-	-	4.90	0.027	4.20 ^b^	4.51 ^a^

^a, b^ Different letters indicate a significant difference (*p* < 0.05).
